# Sea urchin histamine receptor 1 regulates programmed cell death in larval *Strongylocentrotus purpuratus*

**DOI:** 10.1038/s41598-018-22397-4

**Published:** 2018-03-05

**Authors:** Keegan Lutek, Rasmeet Singh Dhaliwal, Terence J. Van Raay, Andreas Heyland

**Affiliations:** 1 University of Guelph, Integrative Biology, Guelph, ON N1G 2W1 Canada; 2 Present Address: Department of Biology, University of Ottawa, Ottawa, ON K1N 6N5 Canada

## Abstract

Settlement is a rapid process in many marine invertebrate species, transitioning a planktonic larva into a benthic juvenile. In indirectly developing sea urchins, this ecological transition correlates with a morphological, developmental and physiological transition (metamorphosis) during which apoptosis is essential for the resorption and remodelling of larval and juvenile structures. While settlement is initiated by environmental cues (i.e. habitat-specific or benthic substrate cues), metamorphosis is regulated by developmental endocrine signals, such as histamine (HA), thyroid hormones (THs) and nitric oxide (NO). In the purple sea urchin, *Strongylocentrotus purpuratus*, we found that suH1R mRNA levels increase during larval development and peak during metamorphic competence. SuH1R positive cell clusters are prominently visible in the mouth region of sea urchin larvae, but the protein appears to be expressed at low levels throughout the larval arms and epidermis. SuH1R knock-down experiments in larval stages show that the function of suH1R is in inhibiting apoptosis. Our results therefore suggest that suH1R is regulating the metamorphic transition by inhibiting apoptosis. These results provide new insights into metamorphic mechanisms and have implications for our understanding of settlement and metamorphosis in the marine environment.

## Introduction

Marine invertebrate phyla display a diverse array of life history strategies. Many of them develop to adulthood via a distinct larval stage, which transitions into the juvenile through a dramatic metamorphosis. In numerous marine invertebrate groups, settlement defines the transition from the planktonic to the benthic habitat^1^. While settlement is typically initiated by highly specific environmental cues (chemicals from the adult habitat, benthic substrate cues, physical cues etc.), metamorphosis (the morphological, physiological and developmental transition) is regulated by hormone and neurotransmitter systems. For example, in indirectly developing sea urchins a broad array of endocrine and neuroendocrine molecules has been shown to affect metamorphosis. These include dopamine^2,3^, L-DOPA (L-3,4-dihydroxyphenylalanine), glutamine, glutamic acid^2,4^, nitric oxide^5^, thyroxine^6^ and histamine (HA)^7^. Still, the molecular, cellular and developmental mechanisms underlying these life history transitions remain largely unexplored.

Programmed cell death (apoptosis) during normal development is integral to the formation and maintenance of tissue^8,9^, and some of the earliest demonstrations of apoptosis in development come from studies of the metamorphic transition of amphibians and insects (reviewed in^10^), where larval structures, such as the tadpole tail in anurans, are removed while adult structures emerge^11^. Similarly, extensive cell death in larval tissues during echinoderm metamorphosis leaves the juvenile rudiment to develop into an adult; however, few studies have looked at the mechanisms and timing of apoptosis during sea urchin development^7,12,13^.

HA has several putative developmental functions in the purple sea urchin *Strongylocentrotus purpuratus*. In the egg, HA signals via sea urchin histamine receptor one (suH1R) to maintain Ca^2+^ flux during fertilization^14^. This process is mediated via nitric oxide signalling. Furthermore, we previously proposed a dual function of HA in late larval stages^7^. In pre-competent larvae (larvae that are not yet able to respond to an appropriate settlement cue), HA appears to aid in the attainment of metamorphic competence^7^. For example, when a set of larvae with a low level of competence are treated with HA, and then induced to settle, a higher percentage of larvae settle compared to larvae that were not treated with HA. In contrast, HA appears to act as an inhibitor of settlement in competent larvae. When competent larvae are exposed to HA and then induced to settle, a lower percentage of larvae settle compared to larvae not treated with HA. In addition, treatment with a mammalian histamine receptor three (H3R) antagonist induced spontaneous settlement and arm retraction (a phenotype indicative of the initiation of metamorphosis) in competent *S. purpuratus* larvae, while treatment with a mammalian histamine receptor one (H1R) antagonist elicited significant arm retraction, but not settlement. Further, the mechanism underlying arm retraction appears to be caspase mediated^7^. These data suggest that the histamine signalling system is essential to the maintenance of metamorphic competence.

Based on immunostaining it is evident that HA is synthesized in a subset of larval cells^7^. Specifically, histaminergic cells are found in the lateral arm clusters (important ganglia implicated in sensory perception) and the apical organ (the “central nervous system” of these larvae – implicated in sensory perception as well as settlement and metamorphosis)^15^.

While we are beginning to understand some of the physiological and developmental effects of HA in larval *S. purpuratus*, we currently lack detailed insights into the mechanism of action of this transmitter. SuH1R is a seven transmembrane g-protein coupled receptor, similar to the histamine receptors of vertebrates, and is the best characterized histamine receptor in the *S. purpuratus* genome^7,14,15^. Thus, as suH1R is similar in structure to vertebrate histamine receptors (which mediate a variety of processes, notably inhibition of apoptosis)^16,17^, and based on the aforementioned pharmacological evidence^7^, we hypothesize that suH1R regulates apoptosis in *S. purpuratus* as well. We tested this hypothesis by analyzing the expression patterns of suH1R throughout larval development and metamorphosis. Knock-down studies of suH1R suggests that suH1R functions in metamorphic competence by inhibiting apoptosis.

## Methods

### Animal Husbandry

Adult *S. purpuratus* (sourced from Point Loma Marine Invertebrate Lab, Lakeside, California) were kept at the Hagen Aqualab (University of Guelph, Guelph, Ontario) on a 16:8 light cycle in recirculating artificial seawater at 11 °C. Urchins were fed ad libitum with *Kombu spp*. three times a week. To induce spawning, adult urchins were gently shaken and/or injected with 0.5–1.0 mL 0.5 M KCl.

### Larval culturing

Following spawning, eggs were fertilized with diluted sperm and checked for fertilization percentage. Once fertilization success was >80%, fertilized eggs were transferred to a 1 L beaker filled with 600 mL of filtered artificial seawater (FASW) and allowed to hatch and develop for two days. At this point, larvae were transferred to 2 L glass beakers filled with FASW at a density of 1 larva/mL. These beakers were kept at 11 °C in a stirring rack (15 RPM) to ensure larvae stayed suspended in the water column. Larval cultures were cleaned and fed ad libitum with *Rhodomonas spp*. three times each week. Cultures were kept on a rotating schedule to ensure that larvae of the appropriate age were available at all times.

### Antibody generation and validation

**Figure 1:**
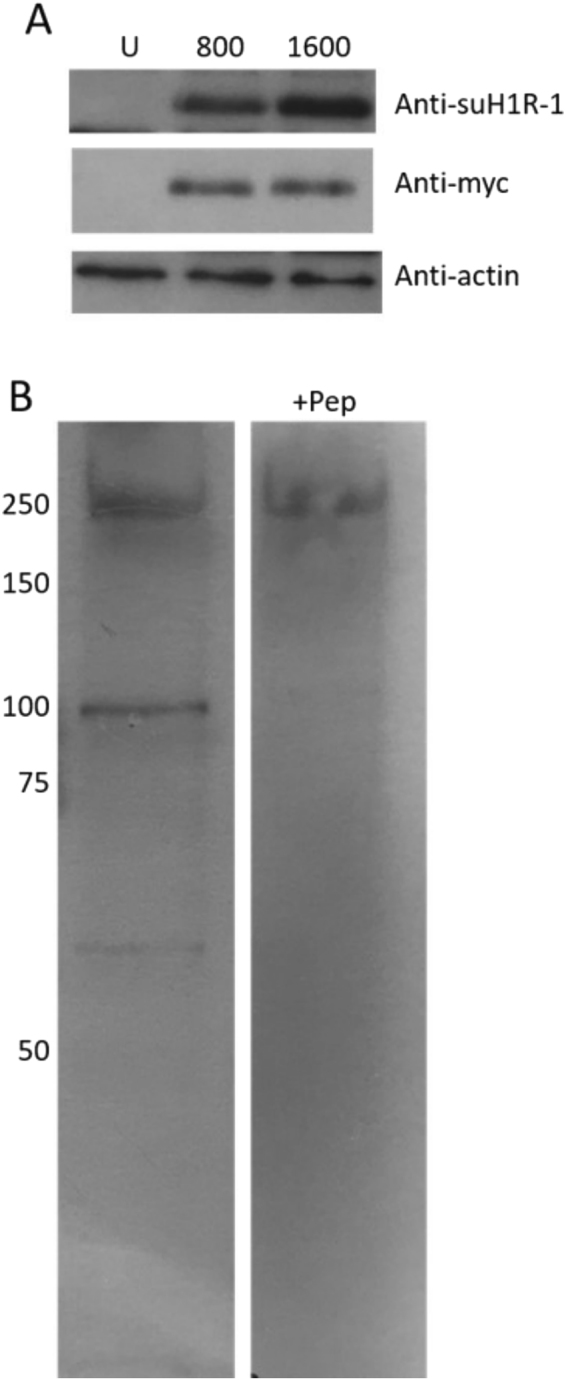
SuH1R antibody. (**A**) A fragment of the suH1R gene containing the suH1R-1 epitope was tagged with 6 × myc tags and 800 pg (lane 2) or 1600 pg (lane3) of mRNA was expressed in zebrafish eggs for protein production. Uninjected (U) eggs served as a negative control and anti-myc served as a positive control. Actin served as a loading control. Both the anti-myc and anti-suH1R-1 antibodies detect a band of ~75 kD, which is the predicted size of the construct. (**B**) The equivalent of 10 competent stage *S. purpuratus* larvae were loaded per lane and probed with the suH1R-1 antibody, which detected 3 bands. All but the high molecular weight 250 kD band disappear by the pre-incubation of the antibody with the immuno-peptide (+pep). The predicted size of suH1R is 76.4 kD. For 1 A original gels of which cropped versions are represented are provided in the Supplement. For 1B both extracts were exposed for the same amount of time.

We used a suH1R specific antibody developed by Gary Wessel^14^ and later developed two new custom affinity purified antibodies. The specificity of the original antibody has been confirmed using Western Blots and several physiological and functional assays in previous publications^14^. The two custom affinity purified suH1R antibody were generated by GenScript in order to validate earlier results with the original antibody used to detect suH1R protein. The peptide sequences identified were AKRIKKGEVDHQLKC (suH1R-1) and CSEFLRDRIKRFSLNKE (suH1R-2). These sequences were identified from the Sp_H1R protein sequence (NP_001012721) aa337–350 and aa564–597 respectively. The suH1R-1 sequence lies within the peptide sequence used for the original antibody generation and applied in previous studies^7^ while the suH1R-2 sequence was modeled after a human H1R antibody. Two New Zealand Rabbits were immunized and antibodies were affinity purified. The specificity of the antibody was tested using IHC and Western Blots (Fig. 1) with pre-immune serum and pre-absorbed peptide as controls. Note that we were only able to validate suH1R-1 using Western Blots (see details below and the Supplement) and therefore did not present any data on suH1R-2. Note that for the results presented here we refer to suH1R-1 as suH1R, as it is based on the same epitope as the original suH1R antibody.

A 1,250 bp partial fragment of the sp-H1R sequence containing the two epitopes was cloned into the pCS2 vector containing 6 N-terminal Myc tags using standard techniques. 800 pg of mRNA generated from this plasmid was injected into a one-cell stage zebrafish embryos and the whole cell lysate from 10 embryos was collected 6 hours post injection into 100 uL of standard RIPA buffer (10 mM Tris, pH 8.0; 1 mM EDTA; 1% Triton, 0.1% sodium deoxycholate; 0.1% SDS, 140 mM NaCl) containing Protease Inhibitors (PI, Sigma S8820). Prior to loading, proteins were denatured by boiling for 5 minutes in 2× sample loading buffer (Final: 0.5 M Tris pH 6.8; 3% SDS; 10% gylcerol; 5% b-mercaptoethanl and 0.002 Bromophenol blue). The equivalent of one embryo per lane was run on a 7% SDS denaturing polyacrylamide gel. Uninjected embryos were used as a negative control. 20 competent *S. purpuratus* larvae were lysed in 50 uL of RIPA + PI and 10 uL of 5 × Sample buffer and divided between two SDS denaturing polyacrylamide gels. The anti-myc (sera from cloned cell line) and suH1R-1 was used at 1:1000 in 1 × TBST (Tris Buffered Saline with 0.1% Tween 20) with 5% non-fat dry milk as a blocker. Secondary antibodies were HRP-conjugated anti-Rabbit (suH1R-1 and suH1R-2) or anti-mouse (anti-myc) used at 1:1000 in 1 × TBST with 5% non-fat dry milk. To block the antibodies, they were pre-incubated for 3 hours at RT with 20ng/ul of respective peptide used to generate them before they were added to the blot. Note that all experiments were performed in accordance with relevant guidelines and regulations. All experimental protocols were approved by the University of Guelph animal care committee (protocol 3614 to TJVR).

### Immunohistochemistry

For immunohistochemistry, larvae were fixed in 4% paraformaldehyde (PF) according to age: <1 week – 15 minutes, 1–2 weeks – 30 minutes, 2–3 weeks – 45 minutes and 3 + weeks – 60 minutes. The procedure for IHC (whole mount immunohistochemistry) was performed according to Sutherby *et al*.^7^ with the following modifications: specimens were initially rinsed 6 times in PBST (0.3% Triton-X in 1× phosphate buffered saline (PBS)) for 10 minutes each, block time was 30 minutes, specimens were washed 5× following primary antibody incubation, secondary antibody incubation was at 8 °C, specimens were washed 5 times in PBST following secondary antibody incubation and larvae were incubated at room temperature in a 1:5000 dilution of Hoechst (a nuclear stain) in PBS for 15 minutes in the dark. The suH1R antibody was used at a concentration of 1:80. suH1R-1 was used at 1:1000. Following immunostaining, larvae were mounted in DABCO (1,4-Diazabicyclo[2.2.2]octane)-glycerol mounting medium (90% glycerol, 0.1 M Tris-HCl, 10 g/L DABCO). After mounting, larvae were imaged on a Nikon Ti inverted microscope and on a Leica confocal microscope (CLSM SP5). Images were then processed for presentation or cell counts in ImageJ.

### qRT-PCR Experiments

#### RNA Extraction and qRT-PCR

RNA was extracted from whole larvae using the Direct-zol^TM^ RNA MiniPrep kit (Zymo Research #R2050; Lot #ZRC186120) according to the manufacture’s instructions. The quality and concentration of the RNA was assessed using a Nanodrop 8000 Spectrophotometer (Thermo Scientific). RNA was then converted to cDNA using the Applied Biosystems cDNA synthesis protocol according to the manufacture’s instructions. The cDNA was then stored at −20 °C until it could be used for qRT-PCR.

All qRT-PCR experiments were performed using a StepOne Plus with the standard ΔΔCt method and the “Fast” SYBR green cycle with a ramp rate of 100% (see the Supplement for details). Amplification efficiency for the control and target genes was assessed based upon the melt curves and trial qRT-PCR runs with sequential primer concentrations. Gene primer sequences can be found in the Supplement. We ended up using all primers at 300 nM in a reaction volume of 20 µL.

#### suH1R and HDC Expression Time Series

In the first set of experiments, we established base line expression patterns of suH1R and the putative HA synthesis enzyme – histidine decarboxylase (HDC)^7^ throughout development. Samples were taken at 2 days, 1 week, 1.5 weeks, 2.5 weeks, 3.5 weeks and 4.5 weeks post fertilization. Samples taken at 2 days to 1.5 weeks were 300 larvae per sample, those taken between 2.5 to 3.5 weeks were 100 larvae per sample and those taken at 4.5 weeks were 20–50 larvae per sample. RNA was extracted, cDNA made and qRT-PCR performed, as above, for both suH1R and HDC, with Ubiquitin as the control gene. The two-day sample was used as the reference sample.

Larvae expected to be competent were taken from a batch of 47day old larvae. Their level of competence was determined to be about 30% after induction with 80 mM KCl for 1 hour and a 12 hour recovery period in FASW. Before induction, 3 samples were taken as a “Competent” sample (20 larvae/sample). 200 larvae from the same batch were then induced as described above. After the recovery period, larvae that had metamorphosed were split into 3 samples (~10 larvae/sample), considered “Juvenile” samples, and the larvae that had not metamorphosed were split into 3 samples (~50 larvae per sample), considered “Not Competent” samples. RNA extraction, cDNA synthesis and qRT-PCR were then performed as described above for both suH1R and HDC with Ubiquitin as the control gene. Three samples (100 larvae/sample) of 2 week old, pre-competent larvae were used as the reference sample for the induction experiment.

Finally, the arms of 70 pre-competent larvae (1.5 months post fertilization) were isolated from the body using micro scissors. This isolation was done in seawater and the arms and body were transferred immediately to separate tubes filled with 350 µL TRIzol on ice. RNA extraction and cDNA synthesis was performed as above. The arms sample yielded 25 µL of RNA at a concentration of 4.085 ng/µL, while the body yielded the same volume at 31.26 ng/µL. qRT-PCR was performed as above to determine the level of suH1R and HDC expression with Ubiquitin as the reference gene.

### Vivo-Morpholino Injections

To assess the function of suH1R in larval development, the gene-knock-down experiments must be performed on late larval stages (>30 days of development). In order to avoid perturbation of HA signaling in fertilization and possibly gastrulation^14^ conventional genetic knock-down or overexpression methods are not applicable in our system. However, we have previously validated vivo-morpholino (vMO) injection and soaking experiments for these stages and therefore chose this method for suH1R knock-down studies. vMO probes were designed by Gene Tools based on the published suH1R sequence (LOC503549) (Morpholino sequences can be found in the Supplement). Injections were performed using an injection solution containing a translation inhibiting vMO designed specifically for suH1R, herein referred to as suH1RMO. vMO injections were performed on late stage (>4 weeks) larvae. All injections were performed on a Nikon Ti compound microscope with an attached XenoWorks micromanipulator. Injection needles were prepared from Drummond Microcaps® (Drummond Scientific Company; #1-000-0500) pulled using a Sutter Instrument Flaming/Brown Micropipette Puller (Model P-100). Needles were loaded with 1–2 µL of injection solution and then broken in FASW. Injection volume was set to be as large as possible while not expanding the injected coelomic space. For injection, larvae were placed individually into a 2 cm petri dish filled with 2 mL FASW and immobilized using a suction pipette.

### Vivo-Morpholino validation

To validate vMO specificity and assess efficiency, we 1) injected vMO in late stage larvae and assessed suH1R positive cells using IHC and 2) Bathed larvae in vMO and analyzed suH1R expression using western blot. To determine if vMO injections changed the number of suH1R positive cells as shown by immunostaining, injections were performed with suH1RMO, or a control solution with nuclease free water in the place of the vMO (1:9 dilution of DRAQ5, a nuclear stain, in water or vMO). Manipulation time as well as site of injection was noted for all larvae. Injections were performed on 6 individuals for each treatment, as described above, and these larvae were allowed to recover in a 24 well plate (1 individual/well) overnight. At this point all larvae were fixed in 4% PF for 45 minutes and immunohistochemical detection of suH1R was performed. Larvae were mounted on individual slides in 1,4-diazabicyclo[2.2.2]octane mounting medium and then imaged on the same Nikon Ti microscope used for injections. Additionally, we subjected *S. purpuratus* larvae to a seawater control, control morpholino (100 µM), and suH1RMO (100 µM) overnight at 12 °C and analyzed protein expression levels using Western Blot analysis.

### Morphological Assays

To assess the morphological changes that result from suH1RMO exposure, both pre-competent (~2 weeks old) and competent (~4 weeks old; 9/16 competent = 56%) larvae were soaked for two hours in suH1RMO at different concentrations (50 µM, 100 µM and 200 µM vMO in FASW), control vivo strand morpholino (200 µM control vMO in FASW) or FASW. These larvae were then imaged on a Nikon Ti Compound microscope (z-stack through whole larva) right after soaking, 1.5 hours after soaking and 24 hours after soaking. Larvae were live imaged. Images were processed in ImageJ and approximations of the amount of cell death occurring at each time point were made by measuring the perimeter of each larval arm and counting the number of cells that were dying or being ejected from the larvae. This could then be converted into a number of cells per µm of larval arm perimeter. These measurements were done by the same person performing the experiment, but the identity of the larvae being counted was not known. Measurements of both perimeter and the number of cells were taken only for portions of the arm that were unobstructed in the image.

To determine whether the effects of suH1RMO soaking are affected by excess histamine we exposed pre competent (~2 weeks old) and competent (>60% of larvae settled in response to KCl) larvae were exposed to 10^–6^ M HA or FASW, transferred to suH1RMO (100 µM), control vivo strand MO (100 µM) or FASW for 1 hour or 3 hours and imaged. Larvae were again imaged live, processed in ImageJ and quantified as described previously.

### YO-PRO Assays

In order to determine the type of cell death that occurred following suH1RMO incubation in *S. purpuratus* larvae, we used an assay similar to that of Vega and Thurber^18^. Using the dyes YO-PRO® and propidium iodide (PI) one can differentiate between necrotic and apoptotic cells. Cells undergoing apoptosis display strong YO-PRO® signal while cells undergoing necrosis display both YO-PRO® and PI signal.

To determine whether there was apoptosis during the metamorphic transition of *S. purpuratus* larvae, we exposed competent larvae to either 80 mM KCl (a known chemical inducer of metamorphosis) or FASW. Larvae (n = 5 per treatment) were exposed to their treatment for 20 minutes. Following these treatments, larvae were incubated with YO-PRO® (0.1 µM) and PI (1.5 µM) for 30 minutes and live imaged on a Nikon Ti Compound microscope. Images were then quantified in NIS Elements (Nikon). The number of apoptotic cells was approximated using the bright spot detection function. Parameters for detection were set to capture the majority of strong signals in cells for KCl treated larvae as these larvae exhibit a phenotype known to be associated with a high level of apoptosis (typical diameter: 5 µm, contrast: 400). Bright spot detection was performed for each optical section through the larvae. The number of spots were divided by the cross-sectional area of the widest portion of the larva to account for differences in size between individuals (see the Supplement for an example of bright spot detection). Based on preliminary analysis, the level of necrosis was low for all larvae. Pearson’s coefficient for colocalization of YO-PRO® and PI signal in larvae was used as a qualitative assessment of that low level of necrosis (see the Supplement 6 for correlation values).

To asses the effect of suH1R knockdown on apoptosis, competent larvae were exposed to vivo strand control morpholino (100 µM), suH1RMO (100 µM), suH1RMO (100 µM) and HA (10^−6^ M) or just HA (10^−6^ M) to determine whether HA could reverse the suH1RMO phenotype described in the morphological assays. Following these exposures, half of the larvae were exposed to 80 mM KCl for 20 minutes and the other half was placed in FASW. This would assess whether an external induction cue was necessary to induce apoptosis in competent larvae. Five larvae were used for each combination of initial exposure and subsequent induction. All larvae were imaged and analyzed as above.

### Statistical analysis

suH1R labelled cells and gene expression data were fitted with regression lines in R 3.1.2^19^. ANOVA tests and Tukey HSD post-hoc tests were used to analyze all assay and qRT-PCR data and all graphs were created in R 3.1.2^19^. qRT-PCR data was log transformed when necessary. Data for the morphological assay was transformed using a percentile rank, inverse normal transformation^20^. Data was log transformed for the YO-PRO assay when necessary. Tests were considered significant if p < 0.05. Means are reported + /− the standard error. ${\rm{\Delta }}\bar{x}$ refers to the difference between two group means and is reported + /− the adjusted 95% confidence interval for pairwise Tukey HSD comparisons.

## Results

### Sea urchin histamine receptor 1 is ubiquitous with focal concentration around the mouth region

We generated several antibodies to recognize suH1R. Here we focus on the most robust antibody, suH1R-1 (Fig. 1 – also referred to as suH1R from here on forward). Using a cloned, myc-tagged fragment of the suH1R we first confirmed that the suH1R-1 antibody can detect exogenously expressed H1R (Fig. 1A). Next, we analyzed the whole cell lysate from competent *S. purpuratus* larvae, and found that suH1R-1 detects two discreet bands at ~55 and ~100 kD, along with an indiscreet mass at ~250 kd. The predicted size of suH1R is 76.4 kD. The bands at 55 and 100 kD were significantly reduced by the addition of the immunizing peptide to suH1R-1, suggesting that the lower 55 kD form may be an alternative splice form (Fig. 1B). Incubation of suH1R antibody with the peptide to suH1R-2 had no effect on the signal (data not shown). Note that as pointed out previously, suH1R protein likely migrates differently in Wester Blots from its predicted size due to post-translational modifications^14^.

**Figure 2:**
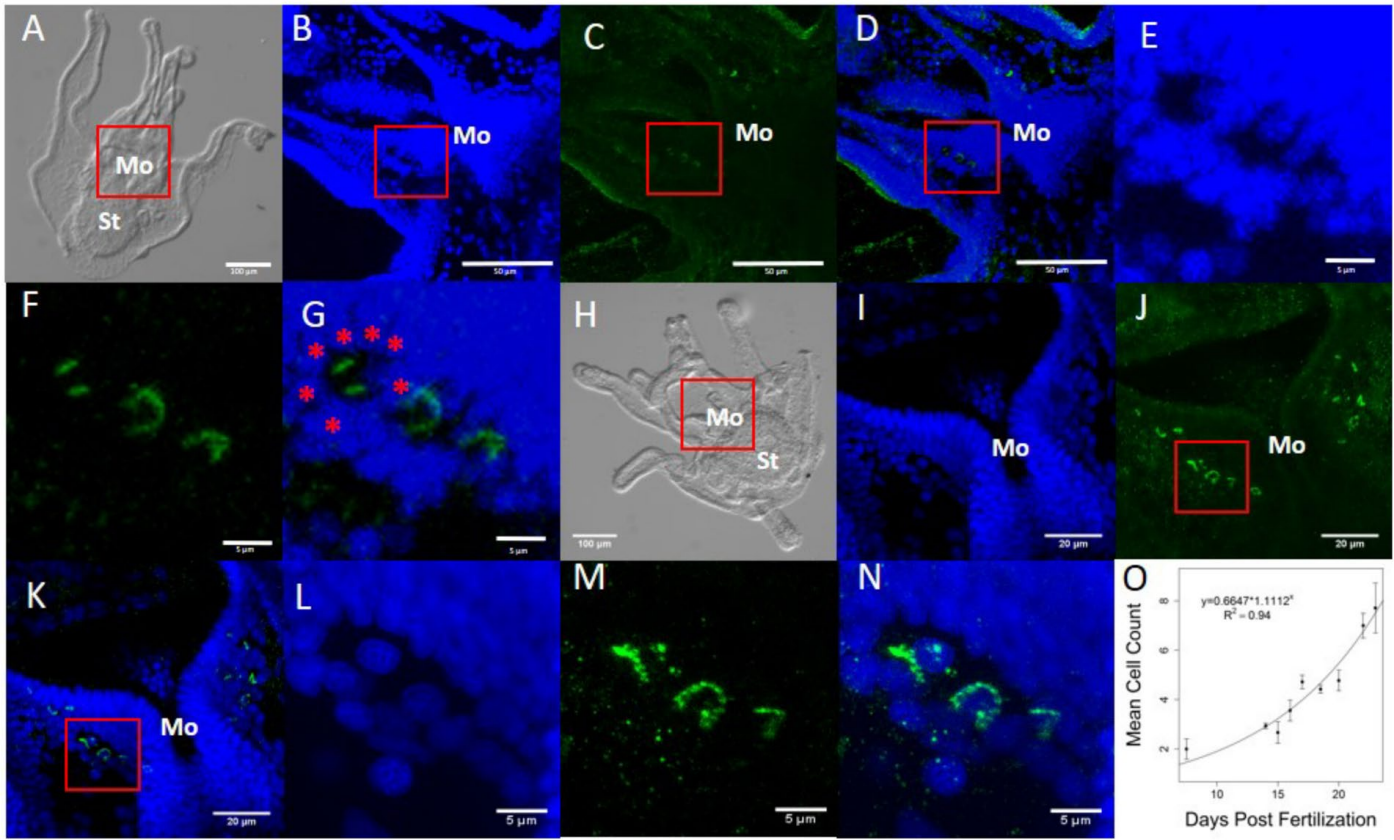
suH1R expression in early (**A**–**G**) and late (**H**–**N**) stage *S. purpuratus* pluteus larvae (suH1R: green; nuclear stain: blue) using compound (**A**,**H**) and confocal (**B**–**G**; **I**–**N**) microscopy. The average number of suH1R labelled cells on one side of the mouth at different points of larval development were quantified (**O**). Specific staining was found on either side of the mouth in a symmetrical pattern. Note that the cells stained for suH1R are surrounded by a 6–7 cells (e.g. red asterisks in **G**). Red squares indicate the mouth region or specific cells highlighted in subsequent images. (**A** and **H**) whole mount larva under DIC light. (**B** and **I**) DAPI labelled cells in the mouth region of the larvae. (**C** and **J**) suH1R antibody staining in the mouth region. (**D** and **K**) combined image of B and C or I and J, respectively. **(E** and **L**) DAPI labelling around suH1R labelled cells. (**F** and **M**) suH1R labelling in specific cells around the mouth. (**G** and **N**) the combination of E and F or L and M, respectively. (**O**) There is an exponential increase in the cell count until larvae reach competence (at 25–30 days post fertilization). Mo: mouth region. St: stomach. (**A** and **H**) single z-plane DIC image, (**B**–**G** and **I**–**N**) are z-projections through the larvae.

To determine if HA signalling contributes to settlement and metamorphosis, we looked at the distribution of suH1R at different stages of larval development. All larval stages (following prism stage) showed enriched staining symmetrically on either side of the mouth with a suH1R primary antibody. Confocal analysis revealed that this staining occurs a few cell layers below the epidermal layer. These stained cells are surrounded by a consistent ring of 6–7 smaller cells (Fig. 2E,G). By analyzing multiple larval stages we also noticed that the number of cells increased as larvae developed closer to metamorphic competence, from approximately 2 cells on either side of the mouth in pluteus stages to 8 or more cells at metamorphic competence (Fig. 2O). Still, low levels of staining where also observed throughout larval structures in all stages (Fig. 2 – note that this is based on the comparison of staining between suH1R and the controls that were pre-absorbed with specific peptide to the antibody).

### suH1R expression levels increase as larvae approach metamorphic competence

**Figure 3:**
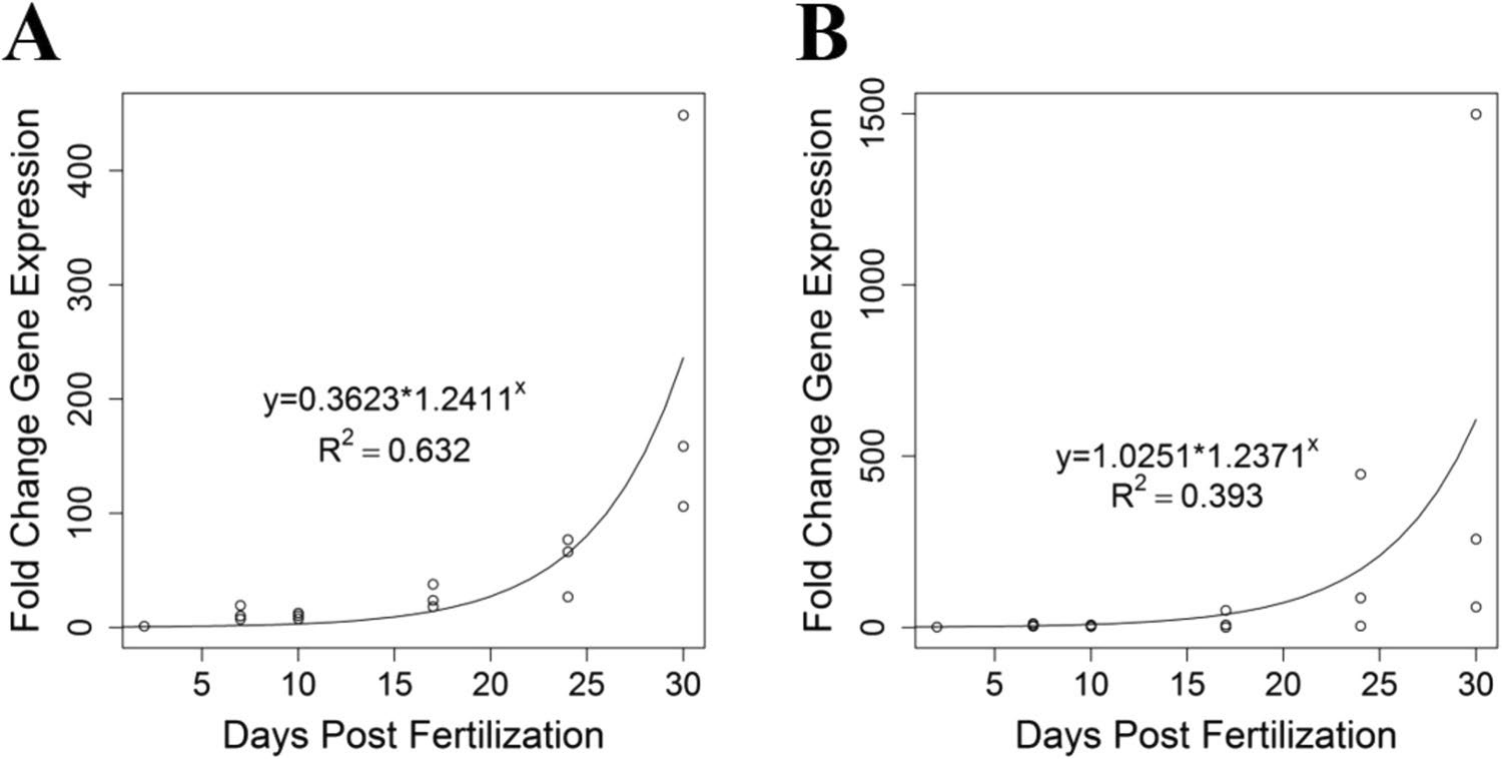
Sea urchin histamine receptor 1 (suH1R; **A**) and histidine decarboxylase (HDC; **B**) expression levels both increase as sea urchin larvae develop towards metamorphic competence. Gene expression levels of both genes were calculated based on three independent biological replicates with two day old larvae as a reference.

To quantify gene expression related to the HA signalling system, we performed qRT-PCR on pluteus larvae (pre-competent and competent larvae) at several ages as well as isolated arms. Larvae were analyzed for suH1R and HDC expression to create a time course of expression for these genes. Expression levels of both HDC (y = 1.025*1.237^X^, R^2^ = 0.393) and suH1R (y = 0.362*1.241X, R^2^ = 0.632) increase exponentially as larval development progresses to metamorphic competence at day 30 (Fig. 3).

**Figure 4:**
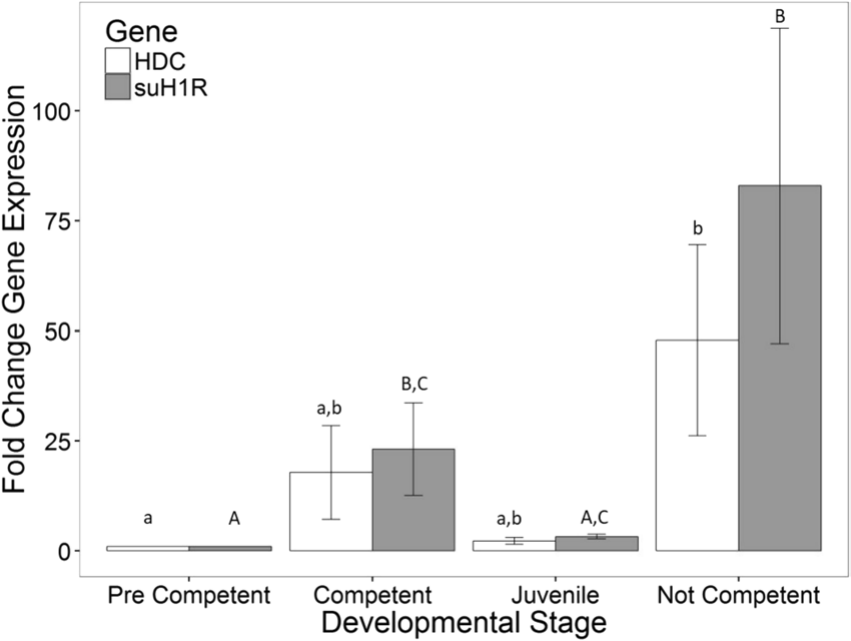
Gene expression levels of sea urchin histamine receptor 1 (suH1R) and histidine decarboxylase for metamorphically competent larvae, juveniles and not competent larvae (which did not settle in response to KCl) relative to two week old larvae. Note an increase in suH1R expression in competent larvae compared to the pre-competent and juvenile samples. Letters denote means that are statistically the same.

If HA signalling is important for the transition between the larval and juvenile phase then we would predict changes in the expression of suH1R and HDC as well. Therefore, we set out to compare the expression of HA genes in pre-competent larvae, competent larvae, juveniles (competent larvae that have been induced to settle) and larvae that were induced to settle, but did not (Not Competent). As above, expression of suH1R increased as pre-competent larvae (n = 3) transitioned into competent larvae (n = 3) (${\rm{\Delta }}\bar{x}$ = 2.82 ± 2.3, p = 0.019). As competent larvae metamorphosed into juveniles (n = 3) there was a decrease in expression of suH1R (${\rm{\Delta }}\bar{x}$ = 1.67 ± 2.3, p = 0.173). However, suH1R expression in larvae that settled (juveniles) was significantly lower than that of their siblings that did not metamorphose when induced to do so (not competent; n = 3) (${\rm{\Delta }}\bar{x}$ = 2.85 ± 2.3, p = 0.018) (Fig. 4). Expression of HDC appears to follow a similar pattern to suH1R expression (low expression in both the pre-competent and juvenile stages and higher expression in competent and not-competent individuals), however, the only statistically significant difference found was between pre-competent (n = 3) and not competent (n = 3) larvae (${\rm{\Delta }}\bar{x}$ = 3.36 ± 2.75, p = 0.019) (Fig. 4). Note that for all time series, the variability in relative gene expression increased at later time points, likely due to greater variability in developmental stage at later time points than is seen at earlier time points.

We also found higher expression of suH1R and HDC in the arms of pre-competent larvae (arms exhibit 1.95 fold higher HDC expression than the body, but 0.585 fold lower suH1R expression than the body for the same total RNA concentration). However, these results may be due to differences in total mRNA between the arms and the body. To account for this we analyzed the threshold values for each part. Body HDC and H1R expression reached fluorescence threshold after approximately the same number of cycles as the arms (C_T_[HDC, body] = 32 cycles, C_T_[suH1R, body] = 29 cycles, C_T_[HDC, arms] = 30 cycles, C_T_[suH1R, arms] = 29 cycles) (Data not shown). Additionally, the control gene used (Ubiquitin) reached the fluorescence threshold after approximately 18 cycles for both the arms and body. These data suggest that suH1R and HDC are likely expressed in cells of the arms as well, despite the fact that protein levels were not detectable using suH1R antibody. Taken together this data clearly demonstrates that there is a strong correlation between loss of HA signaling and settling during metamorphosis and that there is evidence for suH1R and HDC expression in the arms of larvae.

### suH1R is required to inhibit premature settlement

**Figure 5:**
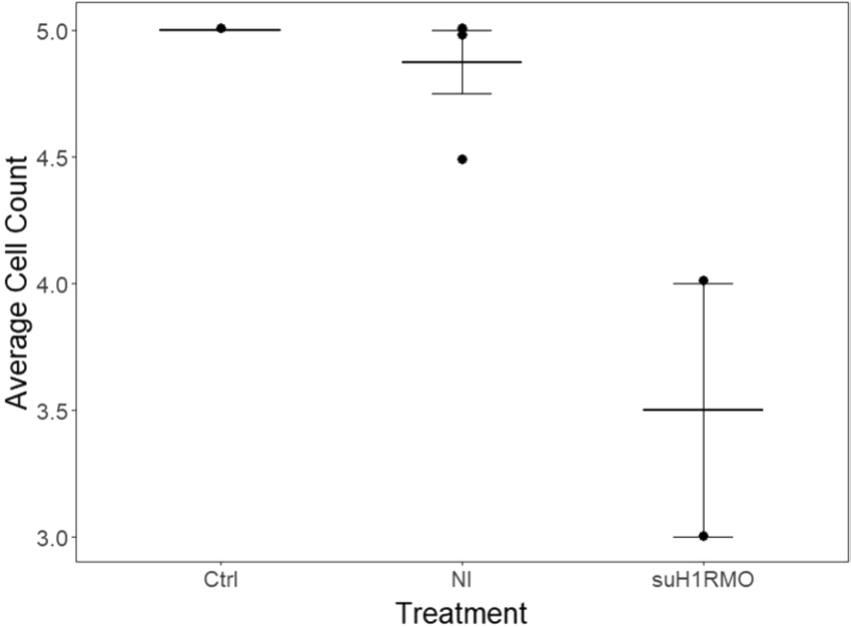
Knock-down of sea urchin histamine receptor 1 (suH1R) decreases suH1R positive cell number in the mouth region of larva. suH1RMO refers to individuals injected with a solution containing a translation inhibiting MO that targets suH1R (22 µM). Ctrl refers to individuals injected with a solution containing nuclease-free water and NI refers to individuals not injected. Due to low sample size no statistical analysis of this data was performed. Quantitative reduction of protein levels is demonstrated by Western Blot (see the Supplement). Error bars represent +/− 1 standard error of the mean.

To determine the role of HA signaling during metamorphosis we needed a method to inhibit its function well after fertilization and gastrulation, and after approximately 4 weeks of development. Clearly, the knockdown of HA signaling classically used to investigate the earliest stages of development (i.e. RNAi or CRISPR/Cas9) would not be appropriate for investigating metamorphosis. Therefore, we chose to use a suH1R antisense oligonucleotide vivo-morpholino (suH1RMO), a methodology for functional knock-downs previously tested in *S. purpuratus*^21^. To demonstrate the efficacy of this MO we performed two tests. First, we used the suH1RMO at late larval stages, which we have previously demonstrated to efficiently knockdown other genes in the developing larvae^21^, and looked specifically at suH1R protein levels by immunohistochemistry. Injection of larvae (>4 weeks post-fertilization) with a 1:9 dilution of DRAQ5 suH1RMO resulted in a reduction of the number of suH1R labelled cells in the mouth region compared to both the injected and uninjected (Fig. 5). Note that we did not perform a statistical analysis of this data as the number of larvae available for analysis was too low. However, we treated competent larvae with suH1RMO (100 µM) and showed that this treatment blocked the expression of the endogenous suH1R protein in comparison to a control morpholino (100 µM) or FASW (Supplement), as demonstrated by Western analysis. Taken together, these experiments demonstrate that the suM1RMO can efficiently inhibit suH1R protein synthesis, while the controls had no effect.

**Figure 6:**
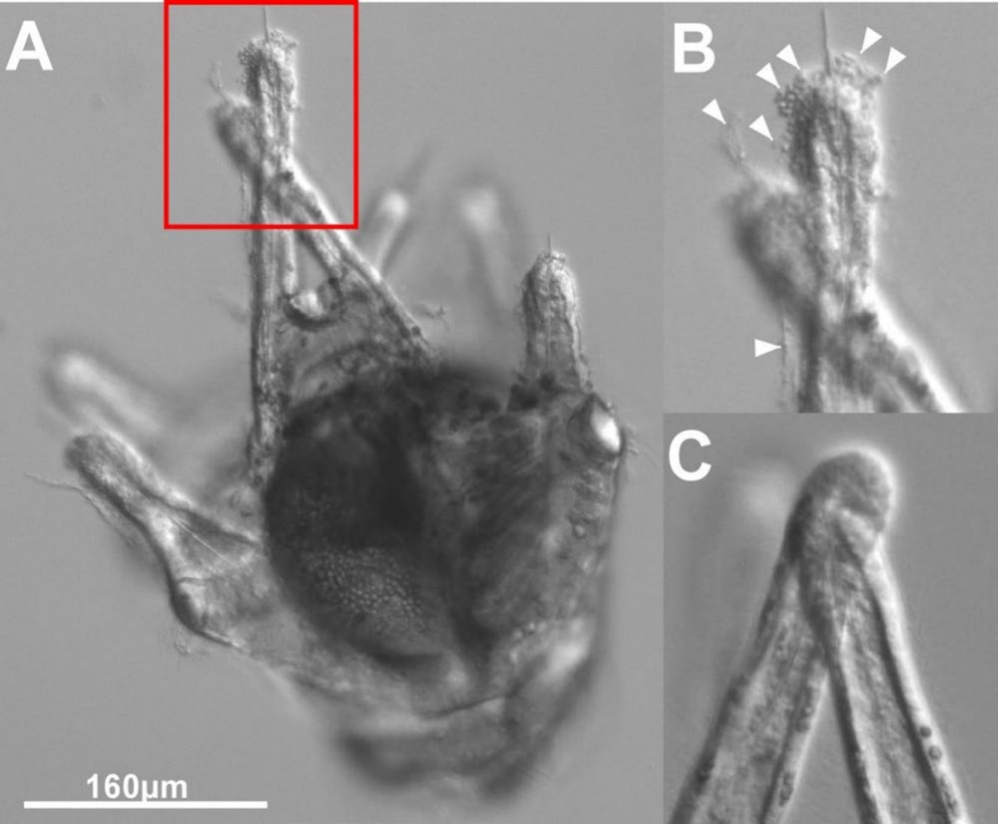
Sea urchin histamine receptor 1 morpholino (suH1RMO) results in cell expulsion at the arm tips of *S. purpuratus*. (**A** and **B**) DIC images of a pre-competent larva treated with suH1RMO. (**C**) DIC image of the arm tips of an untreated larva. Cells at the arm tips appear to be coming away from the rest of the arm tissue in suH1RMO treated larvae, while this phenotype is not observed in untreated larvae. Arrows indicate cells considered expelled or undergoing cell death.

**Figure 7:**
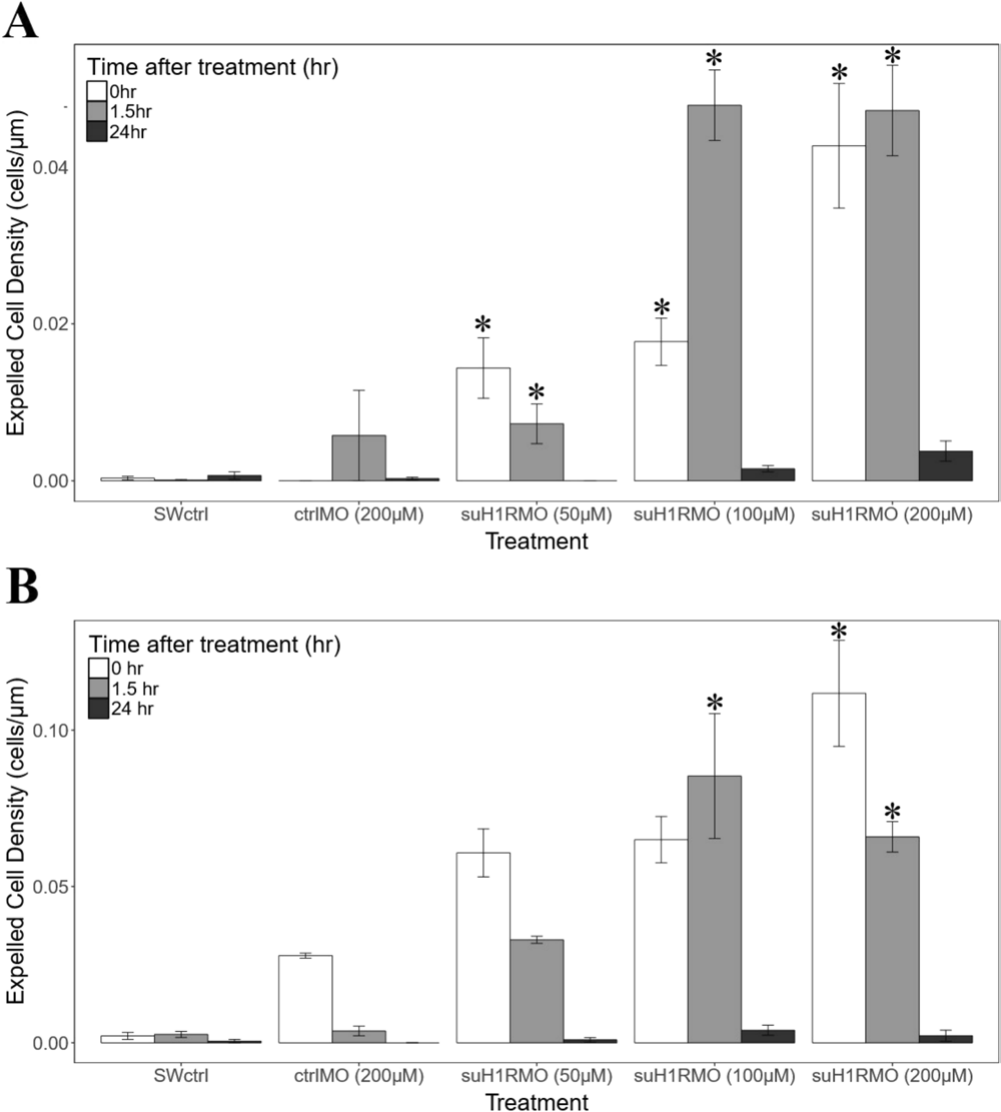
Sea urchin histamine receptor 1 morpholino (suH1RMO) exposure of pre-competent (**A**) and competent (**B**) larvae results in cell expulsion compared to controls. Larvae were soaked in suH1RMO for 2 hours and then transferred to regular seawater. Larvae were imaged directly following soaking (t = 0), 1.5 hours after soaking and 24 hours after soaking. In both competent and pre-competent larvae, the number of cells being expelled per µm of arm perimeter is increased, compared to the control, 1.5 hours after suH1RMO exposure (100 µM and 200 µM) (p < 0.05 in all cases). Increased levels of cell expulsion are also observed for both pre-competent and competent larvae right after the 1 to 2 hour morpholino exposure (t = 0) at the 200 µM concentration (p < 0.05). Additionally, pre-competent larvae have increased levels of expelled cells directly following morpholino exposure at the 50 µM and 100 µM concentrations (p < 0.05). Note that by 24 hours after morpholino exposure, all treatments have levels of cell expulsion that do not differ from that seen in the seawater control (p > 0.05). Asterisks denote a significant difference from the seawater control at the same time point. SWctrl is the seawater control, ctrlMO is the control (random sequence) morpholino, suH1RMO is the sea urchin histamine receptor 1 morpholino. N = 5 for all treatments.

Having established the efficacy of our knockdown morpholino we set out to address the role of HA signaling during metamorphosis. We first treated pre-competent larvae with suH1RMO and observed changes in morphology at several time points, compared to a control MO or seawater alone. We found that pre-competent larvae were rapidly and extremely sensitive to loss of suH1R, exhibiting a higher number of cells being expelled (Fig. 6) from the arms compared to controls. Interestingly, this occurred shortly after the addition of the MO (${\rm{\Delta }}{\bar{x}}_{50\mu M}$ = 0.32 ± 0.26, ${\rm{\Delta }}{\bar{x}}_{100\mu M}$ = 0.36 ± 0.26, ${\rm{\Delta }}{\bar{x}}_{200\mu M}$ = 0.56 ± 0.26; p < 0.01, n = 5 for all concentrations) and for the next 1.5 hours ((${\rm{\Delta }}{\bar{x}}_{50\mu M}$ = 0.29 ± 0.26, ${\rm{\Delta }}{\bar{x}}_{100\mu M}$ = 0.62 ± 0.26, ${\rm{\Delta }}{\bar{x}}_{200\mu M}$ = 0.57 ± 0.26; p = 0.01, p < 0.01, p < 0.01 for 50 µM, 100 µM and 200 µM respectively, n = 5 for all concentrations), and was completed by 24 hours, when cell expulsion had ceased (${\rm{\Delta }}{\bar{x}}_{50\mu M}$ = 0.10 ± 0.26, ${\rm{\Delta }}{\bar{x}}_{100\mu M}$ = 0.14 ± 0.26, ${\rm{\Delta }}{\bar{x}}_{200\mu M}$ = 0.15 ± 0.26; p = 0.98, p = 0.82, p = 0.74 for 50 µM, 100 µM and 200 µM respectively; n = 5 for all concentrations) (Fig. 7A). Further, there was a clear dose dependent effect with 100 µM and 200 µM being more potent than 50 µM (Fig. 7A). We next treated competent larvae the same way and found a nearly identical phenomenon (Fig. 7B). Competent larvae have higher numbers of expelled cells 1.5 hours post 100 µM suH1RMO treatment than the seawater control (${\rm{\Delta }}{\bar{x}}_{100\mu M}$ = 0.39 ± 0.32, p < 0.01, n = 5 for each treatment) (Fig. 7B). Higher numbers of expelled cells are also found just after (${\rm{\Delta }}{\bar{x}}_{200\mu M}$ = 0.60 ± 0.32, p < 0.01, n = 5 for each treatment) and 1.5 hours after (${\rm{\Delta }}{\bar{x}}_{200\mu M}$ = 0.39 ± 0.32, p < 0.01, n = 5 for each treatment) 200 µM suH1RMO treatment of competent larvae compared to the seawater control (Fig. 7B). All treatments did not differ significantly from the seawater control by 24 hours post suH1RMO treatment (${\rm{\Delta }}{\bar{x}}_{50\mu M}$ = 0.06 ± 0.32, ${\rm{\Delta }}{\bar{x}}_{100\mu M}$ = 0.23 ± 0.32, ${\rm{\Delta }}{\bar{x}}_{200\mu M}$ = 0.09 ± 0.32; p = 0.99, p = 0.17, p = 0.99 for 50 µM, 100 µM and 200 µM respectively, n = 5 for all treatments) (Fig. 7B). Control morpholino treated larvae do not differ significantly from the seawater control larvae at any time point (${\rm{\Delta }}{\bar{x}}_{0hrs}$ = 0.20 ± 0.32, ${\rm{\Delta }}{\bar{x}}_{1.5hrs}$ = 0.23 ± 0.32, ${\rm{\Delta }}{\bar{x}}_{24hrs}$ = 0.05 ± 0.32; p = 0.90, p = 0.99, p = 0.99 for just after, 1.5 hours after and 24 hours after treatment respectively, n = 5 for all treatments) (Fig. 7A). This suggests that intact HA signalling, via suH1R, is required to prevent premature or precocious metamorphosis in pre-competent larvae.

**Figure 8:**
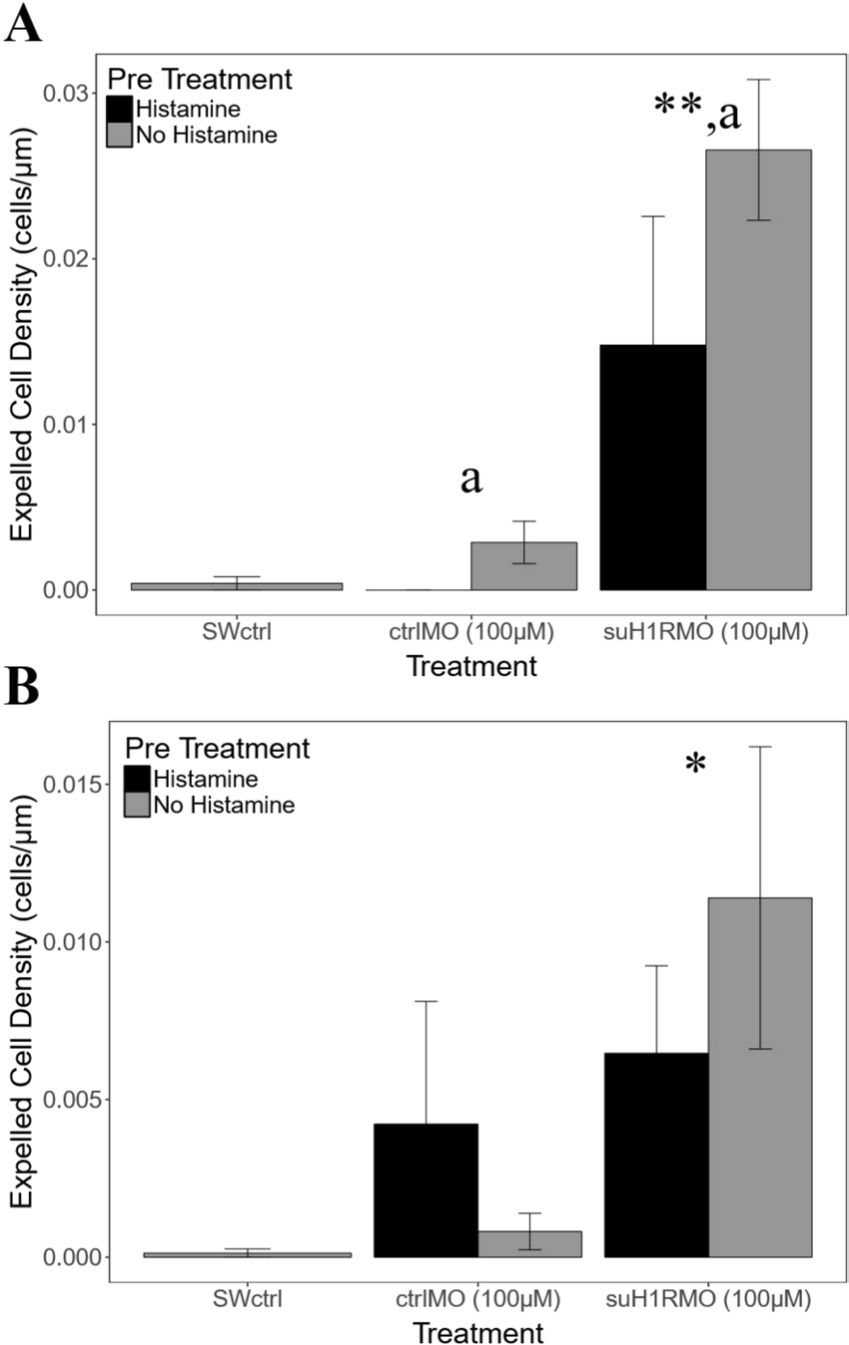
The density of expelled cells along the arms of (**A**) pre-competent and (**B**) competent *S. purpuratus* following a one hour suH1RMO exposure with or without histamine (HA) pre-treatment. Both competent and pre-competent larvae exposed to suH1RMO without HA pre-exposure have a significantly higher number of expelled cells per µm of arm perimeter than the control larvae (in seawater for 3 hours) (p < 0.05). However, when competent larvae are pre-treated with HA before suH1RMO exposure, the number of expelled cells is significantly lower than when larvae are not pre-treated with HA (p < 0.05). *Denotes a significant difference from the seawater control. **Denotes a significant difference between suH1RMO treatment and both, seawater control and control morpholino. “a” denotes a significant difference between larvae pre-treated with HA and those not pre-treated with HA (within a given MO treatment). SWctrl is the seawater control. ctrlMO is the control morpholino. suH1RMO is the sea urchin histamine receptor 1 morpholino. N = 5 for all treatments.

To determine if HA signalling prevents premature metamorphosis we treated pre-competent larvae with HA prior to knocking down suH1R. Pre-treatment of pre-competent larvae with 10^−8^ µM HA did not significantly affect the number of expelled cells (${\rm{\Delta }}\bar{x}$ = 0.03 ± 0.16, p = 0.72, n = 5 for each treatment) (Fig. 8A). However, there were significantly more expelled cells following suH1RMO treatment in pre-competent larvae compared to the seawater control (${\rm{\Delta }}\bar{x}$ = 0.42 ± 0.29, p < 0.01, n = 5 for both treatments) and control morpholino (${\rm{\Delta }}\bar{x}$ = 0.29 ± 0.21, p < 0.01, n = 5 for both treatments) (Fig. 8A). In contrast, pre-treatment of competent larvae with 10^−8^ µM HA significantly reduced the number of expelled cells for larvae treated with both the control morpholino and suH1RMO (${\rm{\Delta }}\bar{x}$ = 0.16 ± 0.09, p < 0.01, n = 5 for all treatments) (Fig. 8B). There were significantly more cells expelled in suH1RMO treated larvae than in either seawater control larvae (${\rm{\Delta }}\bar{x}$ = 0.40 ± 0.15, p < 0.01, n = 5 for both treatments) or control morpholino larvae (${\rm{\Delta }}\bar{x}$ = 0.35 ± 0.12, p < 0.01, n = 5 for both treatments) (Fig. 8B).

### Cells in the arms undergoing retraction undergo apoptosis

**Figure 9:**
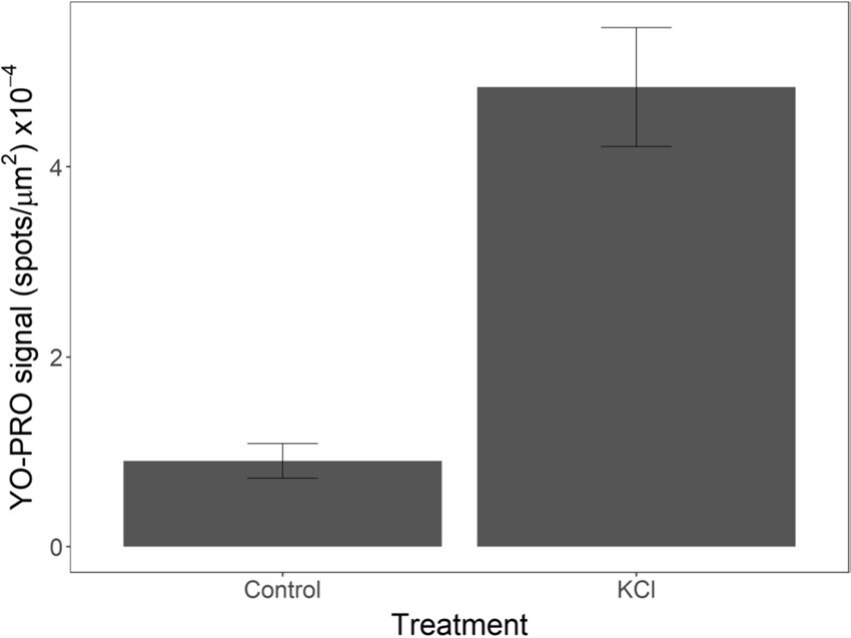
Competent larvae treated with 80 mM KCl exhibit significantly higher YO-PRO signal than untreated (control) larvae (p < 0.05; log-transformed YO-PRO signal). YO-PRO signal was quantified as the number of bright spots detected for each optical slice through the larvae divided by the surface area of the larvae at it’s widest point (Supplement) This value was averaged across all optical sections for each larva. N = 5 for all treatments.

**Figure 10:**
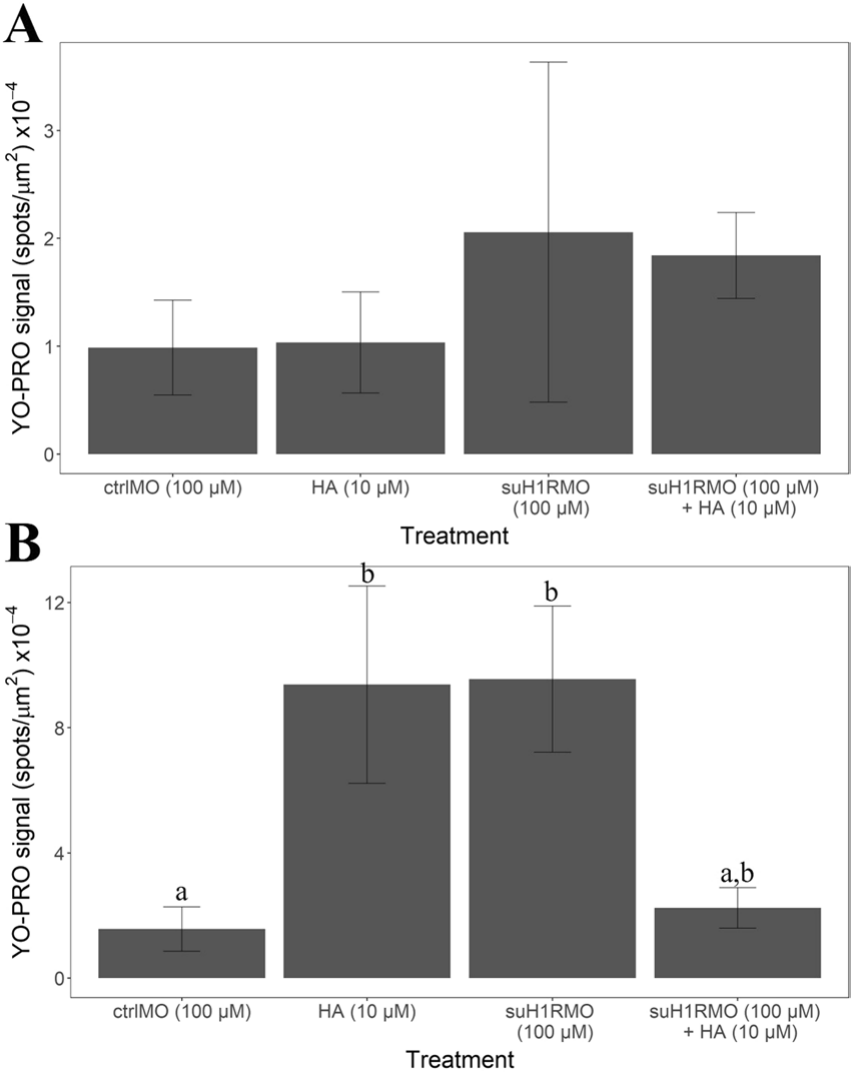
YO-PRO signal for (**A**) competent larvae not treated with KCl and (**B**) competent larvae treated with KCl. There were no significant differences in YO-PRO signal for any larvae not treated with KCl (A; p > 0.05; log-transformed YO-PRO values). YO-PRO signal is significantly higher in histamine treated and suH1RMO treated competent larvae compared to crtlMO following KCl induction (B; p < 0.05; log-transformed YO-PRO signal). ctrlMO is the control morpholino, HA is histamine and suH1R is sea urchin histamine receptor 1. N = 5 for all treatments. Letters denote means that are statistically the same.

The large number of cells being expelled from the arms in competent larvae suggests that the cells are undergoing apoptosis. To investigate this, we induced settlement in competent larvae with 80 mM KCl and used a nuclear stain (YO-PRO®) to investigate apoptosis. As shown in Fig. 9, we observed significantly more YO-PRO® staining in cells treated with KCl than untreated cells (t_8_ = −6.03, p < 0.05). As knockdown of suH1R also results in many cells being expelled from the arms of competent larvae we wanted to confirm if these cells are also undergoing apoptosis. Indeed, we observed a significant increase in YO-PRO® staining in cells treated with suH1RMO compared to a control MO (${\rm{\Delta }}\bar{x}$ = 2.35 ± 1.68, p < 0.01, n = 5 for both treatments), however, this increase is only significant following 80 mM KCl induction (Fig. 10). The number of YO-PRO® positive cells in the suH1RMO + KCl treated larvae was very similar to larvae treated with HA and KCl, which also induces settlement (${\rm{\Delta }}\bar{x}$ = 0.09 ± 1.59, p = 0.99, n = 5 for both treatments). However, as above, we found that pre-treatment of competent larvae with HA offers some protection from suH1R knockdown (${\rm{\Delta }}\bar{x}$ = 1.38 ± 1.6, p = 0.10), although this difference was not statistically significant (Fig. 10B). Importantly, HA treatment had no effect on competent larvae that were not treated with KCl (there were no significant differences between any treatment groups that were not induced with KCl; F_3,16_ = 0.827, p = 0.50) (Fig. 10A). These results suggest that loss of HA signaling induces apoptosis in cells being expelled from the arms as the larvae begins the process of metamorphosis.

## Discussion

Metamorphosis is a rapid process in sea urchin larvae that involves apoptosis and our results suggest that HA signaling functions as a potent inhibitor of apoptosis during this process. Specifically, we have generated an antibody that detects suH1R and showed that suH1R protein is ubiquitously expressed and enriched in putative sensory cells. As larvae undergo metamorphosis we demonstrated that they rapidly lose the expression of HA signaling components. We also found a marked increase in apoptosis in cells undergoing arm retraction and successfully demonstrated that knockdown of suH1R resulted in a rapid expulsion of cells from the arms that undergo apoptosis.

### suH1R acts as an inhibitor of apoptosis throughout larval development

Apoptosis is a key part of embryonic and larval development in sea urchins^7,12,18,22^, and caspase activity has been documented in the metamorphic transition of *S. purpuratus*^7^. In this study we show that suH1R knockdown results in a marked increase of apoptosis in competent larvae based on YO-PRO® staining, and that this effect is partially reversed by HA in competent larvae. Additionally, suH1R knockdown results in an increased number of expelled cells in both pre-competent and competent larvae. Furthermore, we previously showed that treating competent larvae with HA receptor antagonists can increase caspase activity^7^. We view these finding as evidence that suH1R functions as an inhibitor of apoptosis in *S. purpuratus* larvae. Based on our findings that both suH1R expression and the number of suH1R positive cells increase during larval development, towards metamorphic competence, we conclude that suH1R’s function in programmed cell death also plays a key role in the inhibition of apoptosis during metamorphic competence. Interestingly, this is in agreement with previous work on larval *S. purpuratus* which demonstrated increased caspase activity in response to mammalian H1R antagonists^7^.

Competent larvae treated with suH1RMO show a subtle, but important, difference in phenotype in comparison to larvae treated with generic HA receptor antagonists^7^. Specifically, we found that the morpholino induced phenotype is largely confined to the arms of larvae, while antagonist treatment of larvae showed extensive cell death throughout the body and arms, some of which can be due to the cyto-toxic effects of the antagonists^7^. Moreover, none of the larvae treated with suH1RMO exhibited signs of either settlement or metamorphosis, as was also the case with previous pharmacological antagonist treatments, suggesting that although suH1R may be an important part of the metamorphic process, it is not sufficient for settlement and may be an upstream regulator of larval metamorphosis. Our results suggest that suH1RMO exposure results in significantly increased levels of apoptosis only if this exposure is followed by KCl induction. This data also suggests that the suH1R signalling pathway is upstream of an exogenous inducer.

Our results also support previous investigations of apoptosis during sea urchin metamorphosis. We have demonstrated that apoptosis occurs in *S. purpuratus* larvae directly following induction of settlement and metamorphosis by KCl. This result is similar to the observed TUNEL staining during larval arm resorption and metamorphosis in *Hemicentrotus pulcherrimus*, however the apoptosis we observed occurred much faster than that observed in *H. pulcherrimus*^12^. This may be a by-product of the faster metamorphic transition observed in *S. purpuratus* following KCl induction (*S. purpuratus* is fully metamorphosed within 12 hours, while *H. pulcherrimus* takes 24 hours to complete metamorphosis) or, it may be that KCl induces a more rapid metamorphosis than a natural cue would. Unfortunately, the natural cue for *S. purpuratus* has not yet been determined and previous attempts to use biomedia as an inducer had limited success. Note also that TUNEL staining in sea urchins larvae has repeatedly produced results with a lot of background staining in whole mount sea urchin larvae^12,13,22^. We also found that YO-PRO® staining results in much more reliable results that TUNEL. This is likely due to the fact that YO-PRO® can capture very early onset of apoptosis while TUNEL only detects DNA fragmentation, the final stage of apoptosis. We hypothesize that due to the fast progression of apoptosis in sea urchin larvae, most cells from the larval arms that are TUNEL positive are not physically attached to the arms anymore and can therefore not be detected. YO-PRO® staining therefore is likely the better method to detect and measure apoptosis in sea urchin larvae.

As noted earlier, at least one other HA receptor has been previously identified in the genome of *S. purpuratus*^7,15^. In mammals, HA plays a role in many signalling mechanisms and the mammalian ortholog of this second sea urchin receptor may be important in mediating other processes including apoptosis. It is therefore unclear at this point whether suH1R is the only receptor regulating programmed cell death in *S. purpuratus*. We also hypothesize that mapping the distribution of this second receptor in *S. purpuratus* may result in a clearer correlation between HA distribution (specifically in the arm ganglia^7^) and the receptor. Ultimately, characterization of the second receptor in future studies will help clarify the mechanism of action in the sea urchin.

Since the response to suH1R inhibition was similar in both pre-competent and competent larvae, it is likely that suH1R functions as an inhibitor of apoptosis throughout larval development and that HA’s function in cell death pathways is not restricted to settlement. Apoptosis is essential for proper morphogenesis and suH1R may be important in the regulation of such processes during larval development. Given that the phenotype associated with suH1RMO exposure is largely restricted to the arms of larvae at all stages of development we tested, suH1R may be mediating morphological changes in larval arms. Notably, echinoderm larvae respond to changes in food conditions by lengthening or shortening their arms^23^. When in the presence of low food conditions, larvae sacrifice stability in the water column for improved feeding performance by lengthening their arms. Under high food conditions larvae do the reverse. Preliminary data from our lab (Nguyen *et al*. unpublished) show, for example, that HA treatment of early pluteus larvae results in arm elongation. Future studies will have to investigate the link between HA signaling and arm plasticity in *S. purpuratus*.

### suH1R is found in the mouth region of larval at all stages of larval development

HA has been shown to function during the metamorphic transition in a variety of echinoderm species, including *S. purpuratus*, and an extensive histaminergic nervous system has been described in *S. purpuratus* larvae^7,24^. *S. purpuratus* has at least two genes that code for HA receptors in it’s genome. Previous work in this species has only focused on suH1R^14^. Our results show that suH1R protein is enriched in cells in the mouth region at all stages after the prism stage. Our critical and thorough analysis with a new H1R antibody revealed a lot of unspecific binding of the previous antibody, now confirmed by blocking specific binding sites of the antibody with target peptide in this study. It is important to note that the cells expressed in the mouth region likely represent only a small number of the cells expressing suH1R in the larva. For example, our detailed IHC consistently showed light staining in the arms in comparison to pre-absorbed controls and no antibody controls. Therefore, we conclude that the weaker staining in the arms is likely specific but our IHC assay is not sensitive enough to provide sufficient signaling for detailed analysis in late larval stages. This hypothesis is supported by the fact that gene expression levels exceed the levels that could be expected from just a dozen of cells in late larval stages, indicating a larger number of cells in the larva expressing the receptor and synthesis gene. Furthermore, our previous analysis of suH1R expression in early development showed a much broader expression in embryos of the sea urchin^7,14^. Therefore, we assume that the IHC on whole mount late stage larvae likely is not sufficiently sensitive to detect suH1R expression in smaller cells or cells that express less protein. These expression levels were, however, captured with our Western Blot analysis.

In this study, the enriched suH1R positive cells surrounding the mouth appear more elongate than the surrounding cells and are in close proximity to other cells shown to have neuronal functions (i.e. the lateral arm clusters)^15^. However, we did not find any morphological evidence that these cell bodies protrude above the epithelium of the larva. We speculate that these cells are receptor cells, functioning as part of an internal signalling system, receiving input from endogenous cues (via endocrine or paracrine methods) rather than exogenous cues. The clusters of suH1R positive cells in the mouth region are in close proximity to the histaminergic lateral arm clusters, which we previously identified using a specific antibody to histamine^7^. We hypothesize that HA likely diffuses extracellularly throughout the larva and activates receptors near the release site. However, as mechanisms of endocrine signalling are rarely studied in sea urchins [but see^23–28^ for examples of studies that do look at endocrine regulation of echinoderm reproduction], we can only speculate about these signalling mechanisms. We propose and discuss two potential mechanisms here: (1) volume transmission, the diffusion of chemical signals through neural tissue, and (2) diffusion through the coelomic cavity. Future studies will be aimed at determining the true nature of these signalling mechanisms.

Volume transmission has been documented in the soma, dendrites and axon varicosities of leech neurons, where neurotransmitters are released extrasynaptically to permit the transmission of these signals across distances much greater than the synaptic cleft^29^. Since larval sea urchins possess a nervous system with nerve varicosities and ganglia^15^ there is the potential for such a mechanism to occur. Further study of the distribution and release of HA in *S. purpuratus* larvae may help determine if volume transmission does indeed occur in sea urchin larvae.

The body cavity of a variety of larval echinoderms has been shown to consist of a gel-like substance, which could facilitate the diffusion of signals^30^. While the exact chemical properties of this gel remains unknown, the blastocoelar extracellular matrix of *S. purpuratus* is composed of sulfated glycoproteins and sulfated proteoglycans, including dermatan sulfate^31,32^. Given that dermatan sulfate is also the most abundant glycan in mammalian skin and that HA signalling during allergic reactions occurs through this skin, we therefore predict that HA signalling can occur through an ECM that contains dermatan sulfate. Determining the precise makeup of the extracellular matrix in later stage larvae would be a first step towards testing this hypothesis.

### suH1R expression and the histamine signalling system

Our results support the idea that an increase of suH1R expression both aids the attainment of metamorphic competence and maintains metamorphic competence once achieved. Gene expression of both HDC and suH1R increase as competence is achieved, as does the number of suH1R positive cells in the mouth region, and knock-down of suH1R leads to increased levels of apoptosis in comparison to the control treatment (after induction with KCl). This is in accordance with previous investigations of the histamine signalling system in *S. purpuratus* larvae and is also in accordance with investigations of apoptosis throughout sea urchin larval development^22^. It appears that as sea urchin larvae reach competence, the rate of apoptosis decreases. Our results suggest that an increased level of suH1R likely inhibits apoptosis in competent larvae and that a decrease in suH1R levels, or inhibition of its activity, results in apoptosis necessary for the morphological changes associated with settlement. Additionally, the low levels of suH1R following settlement would allow for larval tissues to be resorbed and facilitate proper formation of juvenile structures. Thus, the timing of suH1R and HDC expression appear to mirror those times during *S. purpuratus* development when apoptosis may be most critical.

## Conclusion

The results presented here demonstrate that suH1R is expressed throughout larval development and that expression mirrors expected levels of apoptosis at different stages of development. suH1R expression is low in earlier stages of development, and then increases exponentially as these larvae reach metamorphic competence. Expression remains high during metamorphic competence, thereby inhibiting apoptosis and preventing metamorphosis, or maintaining cell viability, until induction by an appropriate settlement cue, or apoptotic cue. In juvenile *S. purpuratus*, suH1R expression is low, to allow for the completion of morphogenesis. Furthermore, knockdown of suH1R leads to premature apoptosis throughout larval development, but only following the induction to settle. We therefore suggest that the HA signalling system in *S. purpuratus* larvae plays an integral role in the maintenance of metamorphic competence through inhibition of cell death in pre-metamorphic larvae.

## Electronic supplementary material

Supplement
