# Supplementary material for: Sea urchin histamine receptor 1 regulates programmed cell death in larval Strongylocentrotus purpuratus
Source: Sci Rep. 2018 Mar 5;8:4002. doi: 10.1038/s41598-018-22397-4 (PMC5838261; doi:10.1038/s41598-018-22397-4)
Supplement: Supplementary file 1 — Supplement [file 41598_2018_22397_MOESM1_ESM.pdf]

# Sea urchin histamine receptor 1 regulates programmed cell death in larval *Strongylocentrotus purpuratus*

**Keegan Lutek[a,1] Rasmeet Singh Dhaliwal[a], Terence J. Van Raay[a] and Andreas Heyland[a]**

**[a]University of Guelph, Integrative Biology, Guelph, ON N1G 2W1, Canada**

**[1]Present address: University of Ottawa, Department of Biology, Ottawa, ON, K1N 6N5, Canada**

| Step | Temperature (ºC) | Time (sec) | Cycles |
|---|---|---|---|
| Holding Stage | 95 | 20 | HOLD |
| Denature | 95 | 3 | 40 |
| Anneal/Extend | 60 | 30 | |
| Melt Curve Stage 1 | 95 | 15 | HOLD |
| Melt Curve Stage 2 | 60 | 60 | HOLD |
| Step & Hold Ramp | +0.3 | | CYCLE |
| Melt Curve Stage 3 | 95 | 15 | |

**Supplement 1.** Run parameters used for all qRT-PCR runs.

| Gene name | Primer Sequence |
|---|---|
| Ubiquitin | Forward: CACAGGCAAGACCATCACAC<br>Reverse: GAGAGAGTGCGACCATCCTC |
| Sea urchin histamine receptor 1 | Forward: TACTCACCTTCTTCCAACT<br>Reverse: CATGCCGAATACTAAACC |
| Histidine decarboxylase | Forward: GGCGGTCGACAAGTTAACAAAAAAATCTCC<br>Reverse: CGCGGTACCAATATTTGGAGCTGCACCCC |

**Supplement 2.** Gene primer sequences used for all qRT-PCR runs. All primers ordered from Invitrogen.

| Target Genes | Morpholino sequence |
|---|---|
| Sea urchin histamine receptor 1 | 5’ CTAATCCAGACGCCATATTGGAGTC 3’ |
| Random sequence control morpholino | 5’ CCTCTTACCTCAGTTACAATTTATA 3’ |

**Supplement 3.** Vivo-morpholino sequences used for morpholino injections and soakings.

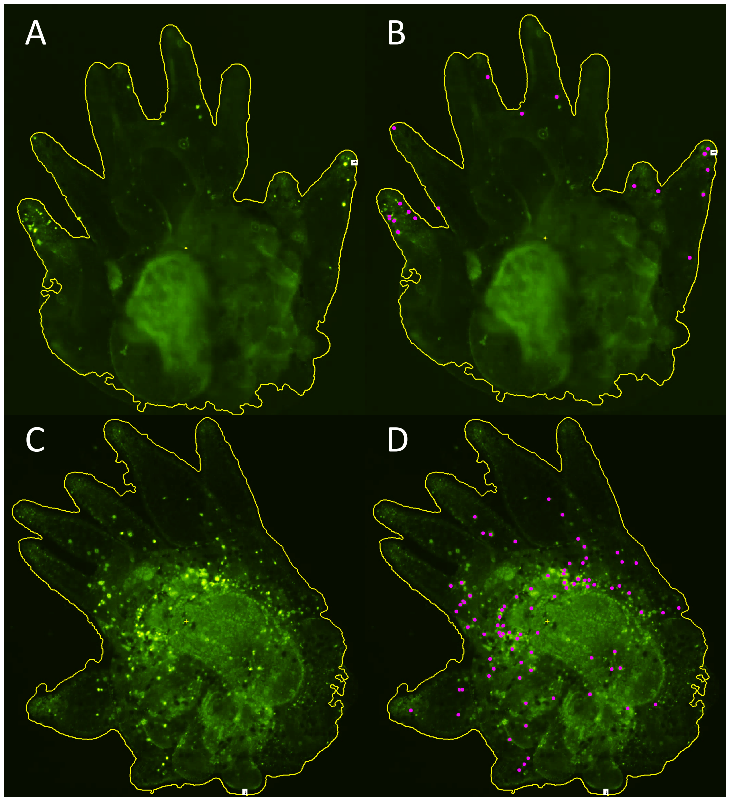


**Supplement 4.** Examples of brightspot detection for control (A and B) and KCl treated (C and D) competent larvae**.** A and C are YO-PRO cannel images without brightspot detection. B and D are YO-PRO channel images with bright spot detection (pink dots represent “bright spots” as identified by the macro in NIS elements). The yellow outline is the region of interest as defined by the auto-detected outline of the larvae from the Hoechst stain channel.

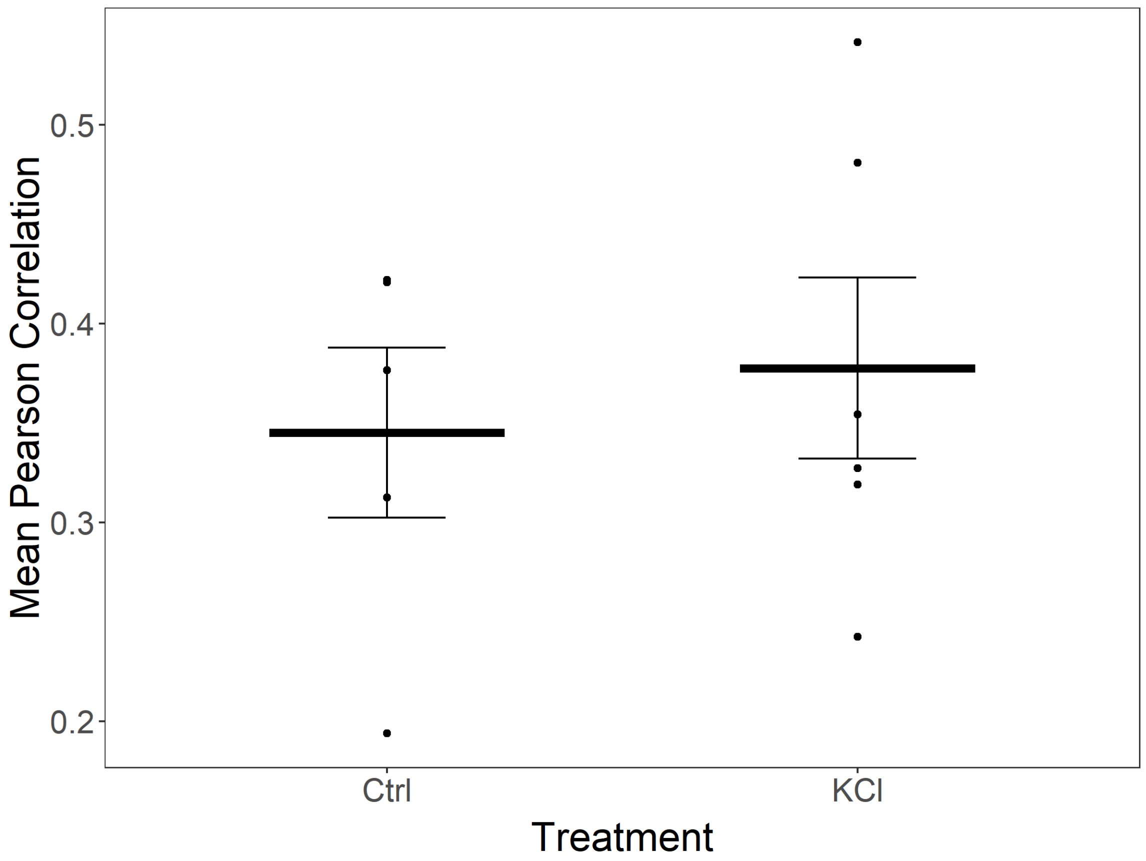


**Supplement 5.** Average Pearson correlation values for the colocalization of YO-PRO and Propidium iodide channels. Pearson correlation was calculated for each optical section through larvae, excluding the stomach area. The stomach was excluded as there is autofluorescence at the PI wavelength in this region and so colocalization including this region might falsely inflate c-olocalization between these two channels.

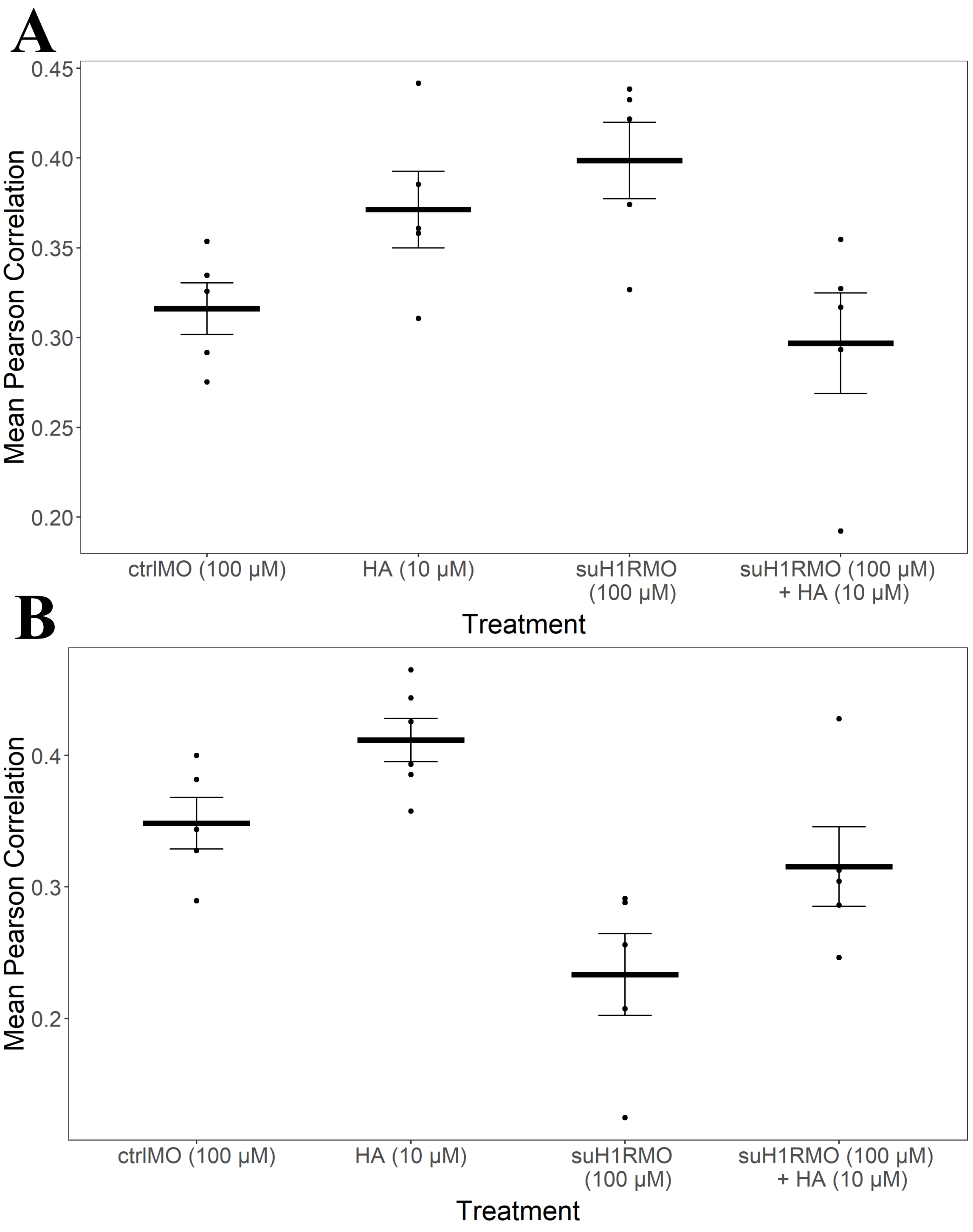


**Supplement 6.** Pearson correlation values for the co-localization of YO-PRO and Propidium Iodide fluorescence in (A) larvae not treated with KCl and (B) larvae treated with KCl. Correlation values are generally low, indicative of little to no correlation between these channels. This suggests that there is little necrosis in any of the treatments.

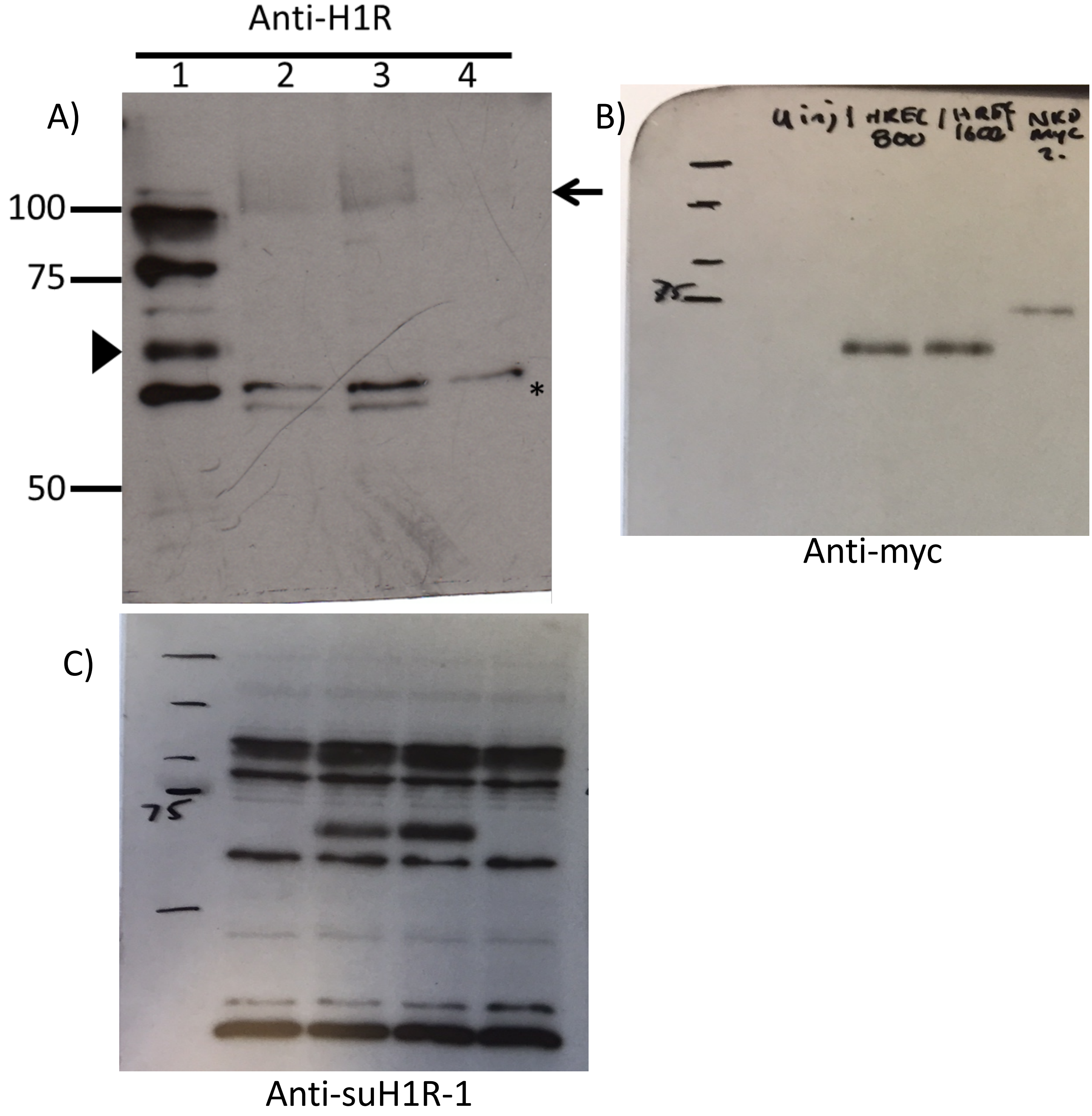


**Supplement 7.** A) H1R Morpholino soaking results in knock-down of suH1R protein. Lane 1- positive control. Whole cell lysate from zebrafish embryo overexpressing a partial fragment of the suH1R-1 receptor (arrowhead). Lane 2- whole cell lysate from *S. purpuratus* larvae treated with sea water; Lane 3- whole cell lysate from larvae treated with control morpholino. Lane 4- whole cell lysate from 15 stage larvae treated with suH1R vivo-morpholino. Note the absence of the ~100 kD band in the suH1R vMO treated embryos (arrow). The non-specific bands at ~55kD act as surrogate loading controls. At this stage a second band appears (noted by the asterisk) which was not previously observed (compare with Figure 1), but this band is also missing in the control lane. B&C) Full blots for Figure 1 in manuscript. The myc antibody clearly labels the His-Rec fragment in lanes 2+3(800 and 1600 pg of injected mRNA, respectively) and the Nkd1-myc (positive anti-myc control) in lane 4. The suH1R-1 antibody detects the exact same bands in lanes 2 +3, which is the His receptor, but not in lane 4. Lane 1 is uninjected control.
